# Measuring parent food practices: a systematic review of existing measures and examination of instruments

**DOI:** 10.1186/1479-5868-10-61

**Published:** 2013-05-20

**Authors:** Amber E Vaughn, Rachel G Tabak, Maria J Bryant, Dianne S Ward

**Affiliations:** 1 Center for Health Promotion and Disease Prevention, University of North Carolina at Chapel Hill, 1700 Martin L. King Jr. Blvd., CB 7426, Chapel Hill, NC, 27599-7426, USA; 2 Prevention Research Center in St. Louis, Brown School, Washington University in St. Louis, 621 North Skinker Blvd., St. Louis, MO 63130-4838, USA; 3 Clinical Trials Unit (CTRU), University of Leeds, Leeds LS2 9JT, UK; 4 Department of Nutrition, Gillings School of Global Public Health, University of North Carolina at Chapel Hill, 2207 McGavran-Greenberg Hall, CB 7461, Chapel Hill, NC 27599-7461, USA

**Keywords:** Feeding, Measures development, Psychometric properties

## Abstract

During the last decade, there has been a rapid increase in development of instruments to measure parent food practices. Because these instruments often measure different constructs, or define common constructs differently, an evaluation of these instruments is needed. A systematic review of the literature was conducted to identify existing measures of parent food practices and to assess the quality of their development. The initial search used terms capturing home environment, parenting behaviors, feeding practices and eating behaviors, and was performed in October of 2009 using PubMed/Medline, PsychInfo, Web of knowledge (ISI), and ERIC, and updated in July of 2012. A review of titles and abstracts was used to narrow results, after which full articles were retrieved and reviewed. Only articles describing development of measures of parenting food practices designed for families with children 2-12 years old were retained for the current review. For each article, two reviewers extracted data and appraised the quality of processes used for instrument development and evaluation. The initial search yielded 28,378 unique titles; review of titles and abstracts narrowed the pool to 1,352 articles; from which 57 unique instruments were identified. The review update yielded 1,772 new titles from which14 additional instruments were identified. The extraction and appraisal process found that 49% of instruments clearly identified and defined concepts to be measured, and 46% used theory to guide instrument development. Most instruments (80%) had some reliability testing, with internal consistency being the most common (79%). Test-retest or inter-rater reliability was reported for less than half the instruments. Some form of validity evidence was reported for 84% of instruments. Construct validity was most commonly presented (86%), usually with analysis of associations with child diet or weight/BMI. While many measures of food parenting practices have emerged, particularly in recent years, few have demonstrated solid development methods. Substantial variation in items across different scales/constructs makes comparison between instruments extremely difficult. Future efforts should be directed toward consensus development of food parenting practices constructs and measures.

## Background

The role of the home environment in shaping a child’s diet and growth is an area of increasing interest, particularly among those working in child obesity prevention and treatment. The home environment has significant influence on child socialization [1], including adoption of eating behaviors [2]. This is particularly true for younger children (2-12 years old) given their limited autonomy and dependence on adult caretakers, who influence dietary intake and eating behaviors through the foods they provide as well as the social environment they create [3].

Parent food practices and feeding style represent a large component of parent behaviors that influence child diet and/or weight. Parent food practices are the specific techniques or behaviors used by parents to influence children’s food intake [4]. Traditionally, food practice constructs have included pressure to eat, restriction, monitoring of the child’s food intake, or the use of rewards for food consumption. More recently, constructs have been expanded to include parent food modeling, family mealtime environments, food preparation practices, involvement of children in food planning and preparation, and control allowed to children over when, where, what and how much they eat. While food practices are specific behaviors or actions, they are often used to categorize parent feeding style [5]. A parent’s feeding style reflects the emotional climate in which these practices occur, or the balance between demanding versus responsive feeding practices [6].

Reviews of family environmental correlates have found fairly consistent associations between child fruit and vegetable consumption and parent food practices such as dietary modeling, food rules, and encouragement [7-9]. However, these reviews have also highlighted gaps in the literature with regard to measurement. How constructs are defined and measured is highly variable across studies, making it difficult to draw clear conclusions. Additionally, studies tend to assess only a limited number of constructs; thus hampering efforts to understand the relative importance of factors and how they might interact. While there have been two recent reviews on measurement of home food availability and accessibility [10,11], there has not been a similar review focused on measurement of the parent behaviors that influence child diet.

This paper addresses this gap in the literature by presenting results from a comprehensive, systematic review designed to identify and evaluate instruments or specific scales assessing parent food practices. It captures the full array of parental food practices thought to shape the sociocultural food environment of the home in an attempt to bring some order to a field of measurement that has become increasingly complex and confusing.

## Methods

**Figure 1:**
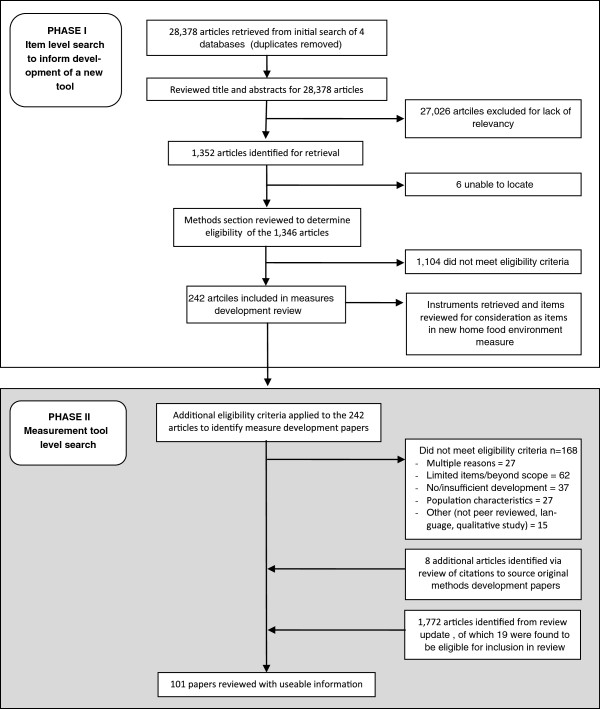
Overview of two-phased literature review.

This review was conducted in two phases (depicted in Figure 1), beginning with an extensive systematic review of the literature to identify factors within the home environment hypothesized to relate to children’s diet and/or eating behaviors. During this first phase, both social and physical characteristics of the home environment and any evidence of their relationship to child diet, eating behaviors, or weight were explored. This initial review was conducted as part of a larger study to identify potential constructs and items for consideration in the development of a comprehensive measure of the home food environment (known as the Home Self-administered Tool for Environmental assessment of Activity and Diet, or HomeSTEAD, R21CA134986). The search terms used and inclusion and exclusion criteria employed reflect this goal. During the second phase, results of the initial review were used to identify articles describing development of instruments assessing parent food practices.

The initial systematic literature review was conducted in October of 2009 using four search engines: PubMed/Medline, PsychInfo, Web of knowledge (ISI), and ERIC. Search terms were identified to capture the following topic areas: (1) home environment or parent behaviors and (2) feeding practices, dietary habits, or eating behaviors. (A detailed description of search terms is available in Additional file 1). No limits were placed on date of publication, but articles had to be in English.

Titles and abstracts were reviewed to narrow results. Percent agreement between reviewers (AV, RT, MB) based on a 5% sample of search results ranged between 93-95%. Disagreements were discussed by all authors; discrepancies were resolved via consensus; and inclusion/exclusion criteria were refined. Following completion of the title and abstract review, full articles were retrieved and reviewed (by either AV or RT) to determine whether or not the paper met the full inclusion/exclusion criteria.

### Inclusion and exclusion criteria

**Figure 2:**
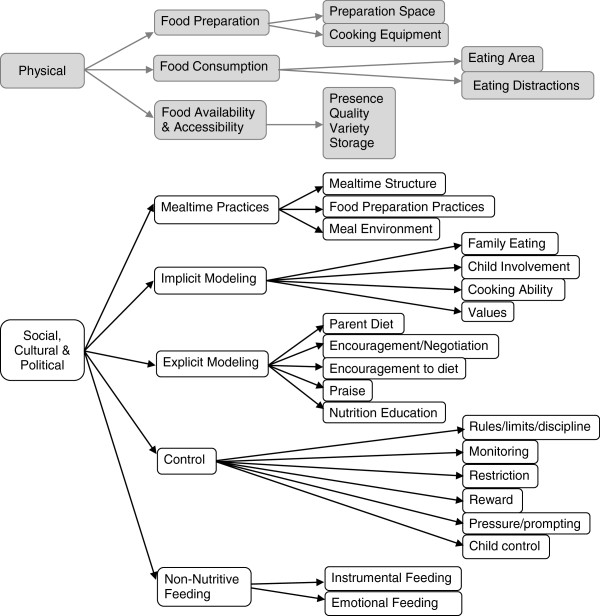
Content map used to guide review.

During the first phase, inclusion criteria specified that the methods section had to describe the measurement of physical and/or social-cultural characteristics of the home environment related to diet and/or eating behaviors in children aged 2-18 years. A content map (Figure 2) based on the ANGELO framework [12] guided the review and ensured inclusion of all relevant topics. The ANGELO framework identifies four types of environments – physical, socio-cultural, political, and economic – which were then conceptualized and defined very specifically to factors coming from within the home environment. The economic environment is often captured by assessing household income, parent occupation, parent education, and similar demographic variables. Identifying demographic surveys was not the focus of this review; therefore, the economic environment was viewed as outside the scope. Additional constructs outside the scope of this review included: individual level determinants of behavior (e.g., knowledge, attitudes, self-efficacy, barriers, food security, acculturation), child eating behaviors (e.g., picky eating), and parent or child dietary intake, food expenditures, time use, body image, and factors not specific to the home (e.g., restaurant meals, purchasing behaviors). While these factors may influence the home food environment, they are not a direct measure of that environment.

Articles were also excluded at this stage if they were not peer reviewed (e.g., editorials and dissertations), if they would not aid in the identification of close-ended items (literature reviews, qualitative studies, case reports), or if they referenced use of an existing measure and offered no further development. In cases where existing and relevant instruments were reused, reviewers verified that the original measure development article had been retained in the original search. Articles could also be excluded if the original measure could not reasonably be obtained (e.g., surveys administered in another language with no translation of items provided within the article, articles published before 1995 that provided insufficient detail to recreate items).

In the second phase, additional selection criteria were added to narrow results to articles that described development and/or evaluation of an instrument assessing parent food practices in families with 2-12 year old children. Parent food practices was defined broadly, based on the original content map, to include constructs related to the home’s social, cultural and political environment around food. Measures of the home’s physical environment (food preparation space, food consumption areas, food availability and accessibility) were eliminated, but have been described elsewhere [10,11].

Articles had to contain details regarding instrument development and/or evaluation. This could include steps such as developing items based on formative data, using cognitive interviews to assess item clarity, engaging experts to evaluate content coverage, and at least one method of reliability or validity testing (e.g. test-retest reliability, internal validity, construct validity, etc.). Measures also had to include at least one relevant scale or theory-generated category of items.

### Data extraction and quality assessment

A data extraction form and quality assessment protocol were developed to facilitate the full appraisal of each measure. While quality assessment protocols of patient-reported outcomes do exist [13-16], review and pilot testing of these tools with papers from this review showed that modifications would be required. Therefore, a new protocol was developed based on common elements from existing protocols and DeVellis’ scale development standards [17]. This process was fully piloted by all authors to ensure accuracy of reporting. Percent agreement between reviewers across items was, on average 83.4% (range: 49.1-100). Additionally, any differences in scoring were discussed until agreement on a final score could be reached. Data extracted, included:

• General descriptive characteristics of the measurement tool: reference, name of measure, purpose, total number of items

• Details about sample used for development: sample size, age range and gender (of children), race/ethnicity, SES, country, completed by parent/child/both, subject burden, translation and/or testing in additional populations

• Content: theory or conceptual model employed, list of scales/categories assessed, number of items in each scale/category

Quality evaluated for the following six key elements:

• Conceptualization of instrument purpose: Instruments were scored 1-4 depending on how clearly the paper conceptualized the purpose of the tool and defined constructs intended to be measured (4 = strongly agree, concepts are named and clearly defined, 3 = agree, concepts are named and generally described, 2 = disagree, concepts only named, but not defined, and 1 = strongly disagree, concepts are not clearly named or defined). Additionally, reviewers captured whether or not a theory or conceptual model helped inform this conceptualization (yes/no).

• Development of item pool: Instruments were scored on how systematic the developers’ process was for developing a pool of potential items, taking into consideration the use of multiple methods (e.g., pulling items from existing instruments, consulting expert opinion, extrapolating from qualitative data, and extracting from the literature) and an iterative process. Scores ranged from 1-3 where 3 = fully systematic processes were used, 2 = systematic process were weak or only used for pieces (but not whole instrument), and 1 = no systematic process used/reported.

• Refinement of item pool: Reviewers extracted information about the methods employed to refine the item pool (e.g., expert review, pilot testing or cognitive interviews with draft instrument, assessment of item performance, and use of exploratory factor analysis. When applicable and available, factor loading were recorded so that they could be compared against generally recognized statistical standards to retain only items with factor loading greater than 0.4 and to address any items with cross-loadings greater than 0.32 [18].

• Reliability: To capture evidence of reliability, reviewers extracted information regarding the evaluation of test-retest, inter-rater, and/or internal consistency testing. Results of test-retest and inter-rater reliability testing, which generally present correlation analysis, were extracted so that results could be compared against generally accepted standards where 0-0.2 indicates poor agreement, 0.3-0.4 indicates fair agreement, 0.5-0.6 indicates moderate agreement, 0.7-0.8 indicates strong agreement, and >0.8 indicates almost perfect agreement [19]. Results of internal consistency, which generally report Cronbach’s alpha, were extracted so that results could be compared against generally accepted standards where 0.6-0.7 is questionable (but often considered sufficient in exploratory analyses), 0.7-0.8 is acceptable, 0.8-0.9 is good, and ≥0.9 is excellent [20].

• Validity: Reviewers extracted information about three types of validity: construct validity, structural validity, and criterion validity. Construct validity was defined as evidence that the new scale(s) “behaves the way that the construct it purports to measure should behave with regard to established measures of other constructs.” (DeVellis, pg. 46) This can include evidence of associations/correlations between the new scale(s) and established measures of general parenting practices, child dietary intake or eating habits, and/or child weight. Evaluation of construct validity could employ simple correlations or t-tests, or more complex methods like regression models. While correlations ≥0.3 are considered acceptable, the significance of results must be interpreted in light of the underlying theory. Evidence of structural validity, specifically results from confirmatory factor analysis (CFA), were extracted so that results could be compared against generally accepted cutoffs for “acceptable” fit indices: maximum likelihood-based Tucker-Lewis Index, Bollen’s Delta, Comparative Fit Index, Relative Centrality Index, and Gamma Hat ≥0.95, McDonald’s Centrality Index ≥0.90, Standardized Root Mean Squared Residual ≥0.08, and Root Mean Squared Error of Approximation ≤0.06 [21]. Evidence of criterion validity was also extracted, generally assessed by correlational analysis between the new scale and a gold standard. The criterion used for the gold standard in this review had to be an objective assessment of food parenting practices (e.g., observation protocols completed by trained research staff).

• Responsiveness: Evidence of responsiveness was also extracted. Responsiveness testing is usually conducted using Effect Size statistics or Standardized Response Means (with values greater than 0.5 considered moderate [22]) or by the Reliable Change Index (with 1.96 considered as a minimally important difference [23]).

An updated literature search was conducted in July 2012 to identify additional measures published since the original search. Given the broad scope of the original search, terms were refined to focus the search on food parenting practices (using the diversity of terms uncovered during the original search) and specifically articles describing the development of measures.

## Results

Results from the four search engines were combined and duplicates were identified and removed, resulting in 28,378 unique titles. Review of titles and abstracts narrowed the search to 1,352 articles, and full articles were located and retrieved for all but six. The initial selection criteria narrowed the search to 242 articles; the additional criteria added in the second phase further narrowed the pool to 74 articles; and a review of citations identified 8 additional papers. These 82 articles described development of 57 unique instruments. The updated search identified 18 additional articles, 14 of which represented new instruments. Table 1 provides a description of each instrument identified and Table 2 describes the development processes employed.

**Table 1:** Description of instruments assessing parental feeding practices (in ascending order by year of publication)

**Author, year and name of instrument**	**# items**	**Relevant**^**1 **^**scales (# items, α)**	**Methods of administration**	**Sample description**
**Jensen (1983)** Family Routines Inventory [24,25]	28	meals (3)	Self-administered paper survey	Parents of children aged 16 years or younger, Black and white, working and middle class
**Stanek (1990)** Eating Environment [26]	18	food-related behavior (18)	Self-administered paper survey	Parents of 2-5 year old children
**Seagren (1991)** Parents’ Behavior and Attitudes Toward their Children’s Food Intake [27]	32	parent’s control of child food behavior (17)	Self-administered paper survey	Mothers of 3-4 year old children, primarily white, low income
**Sherman (1992)** Maternal Feeding Practices Questionnaire [28]	15	pushy feeding practices (15)	Interview and self-administered paper survey	Parents of 0-5 year old children, white and Hispanic populations, low income
**Davies (1993)** About Your Child’s Eating [29,30]	25	positive mealtime environment (5, α = 0.80), parent aversion to mealtime (5, α = 0.70)	Self-administered paper survey	Parents of 8-18 year old children, 50% with cancer or chronic illness
**Crist (1994)** Behavioral Pediatrics Feeding Assessment Scale [31]	35	parent’s feelings/strategies (10, α = 0.74)	Self-administered paper survey	Parents of 1-7 year old children, 50% had cystic fibrosis
**Sallis (1995)** Study of Children’s Activity and Nutrition [32]	135	daily meals eaten together (3), food given as a reward (3, α = 0.59), parenting control of eating (9, α = 0.39)	In-person interview	Parents of 4 year old children, white and Mexican American populations
**Koivisto (1996)** Mealtime Practices [33]	20	prompt and assure (5), feel and play and idol (3), rename (1), instrumental and reward (3), postpone meals (1), praise (1), decide portion (1), put on plate (1), rush and nag (2), avoid (1), child decides portion (1)	Self-administered paper survey	Parents of 2-17 year old children, Swedish population
**Humphry (1997)** Feeding Stories [34]	27	no big deal (9, α = 0.70), avoid mess (11, α = 0.76), nurture vs. independence (7, α = 0.71)	In-person interview	Parents of 4-28 month old children, Black and white, lower education
**De Bourdeaudhuij (1998)** Interactions Around Food [35,36]	30	routines (6), communication (9), rules (7)	Computer- assisted, self-administered	Parents and their children aged 12-18 years old, Belgian population
**Golan (1998)** Family Eating and Activity Habits Questionnaire [37,38]	29	stimulus exposure (8, α = 0.78), eating related to hunger (4, α = 0.86), eating style (13, α = 0.88)	Not specified	Parents of 6-11 year old children, Israeli middle class
**Hupkens (1998)** Food Rules [39]	20	food rules (20)	Self-administered paper survey	Parents of 4-14 year old children, Belgian and German populations, middle to lower SES
**Fisher (1999)** Parental Restriction [40]	6	parental restriction of access to the experimental foods at home (6, α = 0.74-0.81)	Self-administered paper survey	Parents of 3-6 year old children, primarily white
**Carper (2000)** Kids’ Version of the Child Feeding Questionnaire [41]	30	restriction (16, α = 0.60), pressure to eat (14, α = 0.71)	In person interview w/ paper survey	Girls aged 4-6 years old, primarily white, also translated for use in French population [42]
**Cullen (2000)** Parent Food-Socialization Practices [43]	161	expectancies (7, α = 0.79), consequences (6, α = 0.70), discouraging practices (14, α = 0.84), child shopping influence (4, α = 0.67), parent FJV preparation practices (10, α = 0.73), child lunch/snack FJV preparation (4, α = 0.82), child dinner FJV preparation (3, α = 0.84)	In-person interview	Parents of 9-12 year old children, racially diverse, also examined differences across race/ethnicity [44] and evaluated in Czech population [45]
**Neumark-Sztainer (2000)** Project EAT [46-48]	221	parental support for healthy eating (4, α = 0.79), family meal patterns (3, α = 0.73), priority of family meals (5, α = 0.73-0.82), atmosphere of family meals (4, α = 0.73), structure/rules at family meals (5, α = 0.60), parental encouragement to diet (2)	Self-administered paper survey	Children aged 12-16 years old, racially diverse
**Ross (2000)** Family Unpredictability Scale [49]	22	meals (5, α = 0.75-0.88)	Self-administered paper survey	Parents of 2-18 year old children, primarily white, higher income
**Baughcum (2001)** Preschooler Feeding Questionnaire [50]	32	pushing the child to eat more (5, α = 0.70), using food to calm the child (4, α = 0.68), child’s control of feeding interactions (3, α = 0.50), structure during feeding interactions (3, α = 0.37), age-inappropriate feeding (2, α = 0.18)	Self-administered paper survey	Mothers of 23-60 month old children, 56% WIC participants, translated into Spanish [51]
**Birch (2001)** Child Feeding Questionnaire [52-54]	31	perceived responsibility (3, α = 0.88), restriction (8, α = 0.73), pressure to eat (4, α = 0.70), monitoring (3, α = 0.92)	Self-administered paper survey	Parents of 2-11 year old children, primarily white, but also tested in Black [55-57], Hispanic [52,55], Japanese [58], Australian [59], and Hmong [60] samples
**Cullen (2001)** Family and Peer Influences on FJV Intake [61]	160	family FJV normative expectations (7, α = 0.88), parent control (11, α = 0.77), permissive eating (4, α = 0.76), food self-preparation (4, α = 0.76), parent FJV/LFF modeling (15, α = 0.89)	Self-administered paper survey	Children aged 9-12 years old, racially diverse
**Tibbs (2001)** Parental Dietary Modeling Scale [62,63]	6	parent diet modeling (6, α = 0.59-0.74)	self- administered paper survey, and phone interview	Parents of 0-13 year old children, including an African American population [62]
**Tiggemann (2002)** Control Over Child Feeding [64]	7	monitoring (5, α = 0.69), family rules (2)	Self-administered survey	Parents of 5-8 year old children, Australian population
**Wardle (2002)** Parental Feeding Style Questionnaire [65]	27	control over eating (10, α = 0.81), prompting/ encouragement (8, α = 0.74), instrumental feeding (4, α = 0.67), emotional feeding (5, α = 0.83). Note: during pre-testing α’s ranged 0.65-0.85.	Self-administered paper survey	Parents of 3-7 year old children, twins, but also tested in parents of 4-10 year old children from low and high SES [66], and Dutch population [67]
**Bourcier (2003)** Eating for a Healthy Life – Strategies to Influence Eating Behavior [68]	14	reliance on self (4, α = 0.66), pressuring (4, α = 0.52), positive (4, α = 0.63)	Phone interview	Parents of 0-17 year old children, primarily white
**Cullen (2004)** GEMS - Diet-Related Psychosocial Questionnaire [69]	116	low-fat food preparation practices (8, α = 0.66) and high-fat food preparation practices (7, α = 0.58)	Not specified	Parents of 7-10 year old girls, African American
**Melgar-Quinonez (2004)** Child Feeding Strategies [70,71]	12	control (4, α = 0.61), accommodating (3, α = 0.44)	In-person interview	Parents of 36-72 month old children, Latino/Hispanic population, low income
**Vereecken (2004)** Food Parenting Practices [72,73]	43	permissiveness/restriction rules (4, α = 0.71), pressure (5, α = 0.74), encouragement through material reward (3, α = 0.75), verbal praise (2, α = 0.94), encouragement through negotiation (5, α = 0.71), encouragement through rationale (fruit: 4, α = 0.81; veg: 4, α = 0.86), discouragement through rationale (sweets: 5, α = 0.80; soda: 5, α = 0.86), catering on children’s demand (4, α = 0.79), avoiding negative behavior (2, α = 0.82)	Self-administered paper survey	Parents of 2.5-7 year old children, Belgian population
**De Bourdeaudhuij (2005)** Pro Children Project [74]	104	parallel scales for fruit and veg: active parent encouragement (2, α_F_ = 0.83 α_V_ = 0.89), demand family rule (1), allow family rule (1)	Self-administered paper survey	Children aged 10-11 years old, from 5 European countries
**Horodynski (2005)** Child-Parent Mealtime Behavior Questionnaire [75]	44	caregiver’s tendency to get upset with child (4, α = 0.77-0.83), caregiver’s tendency to impose requirements on child’s eating (4, α = 0.68-0.70), caregiver’s emphasis on social interactions during meals (8, α = 0.67-0.73)	Self-administered paper survey	Parents of 11-25 month old children, low-income
**Hughes (2005)** Caregiver’s Feeding Style Questionnaire [6,76,77]	24	parent-centered strategies (12, α = 0.86), child-centered strategies (7, α = 0.71). Note: these two scales were used to score two dimensions of demandingness and responsiveness, which can then be used to categorize feeding style.	Self-administered paper survey	Parents of 3-5 year old children, Black and Hispanic, low income, available in English and Spanish
**Tripodi (2005)** Family Dietary Habits (part of the Italian National Institute of Nutrition) [78]	Not specified	family dietary habits (11)	Self-administered paper survey	Parents of 5-6 year old children, Italian population
**Vereecken (2005)** Social and Environmental Influences on FJV Consumption [79]	127	parallel scales for fruit and veg for: perceived parental behavior (2/2, α = 0.71-0.86), socialization-encouragement (4/4, α = 0.92-0.94), permissive eating practices (4, α = 0.73), obligation rules (5, α = 0.78)	Self-administered paper survey	Children aged 11-12 years old
**Arredondo (2006)** Parenting Strategies for Eating and Activity Scale [80,81]	26	limit setting (2 nutr, 4 PA), monitoring (5 nutr, 2 PA), discipline (3 nutr, 2 PA), control (5 nutr, 1 PA), and reinforcement (1 nutr, 1 PA). Final α’s not reported.	Self-administered	Parents of 5-7 year old children, primarily Latino, survey available in English or Spanish
**Ogden (2006)** Overt and Covert Control [82-84]	9	overt control (4, α = 0.71-0.78), covert control (5, α = 0.79-0.83)	Self-administered paper survey	Parents of 4-11 year old children, primarily white, middle class
**de Moor (2007)** Management Techniques of Feeding Problems [85]	13	positive behavioral support (3, α = 0.67), negative behavioral support (4, α = 0.66) and general management techniques (2, α = 0.58)	Self-administered paper survey	Parents of 18-36 month old children, Dutch population
**Gray (2007)** Parental Attitudes around Feeding [86]	46	parental control (9)	Self-administered paper survey	Parents of 5-8 year old children, 50% Black, and 40% lower income
**Musher-Eizenman (2007)** Comprehensive Feeding Practices Questionnaire [87]	49	monitoring (4, α = 0.78-0.87), emotion regulation (3, α = 0.74-0.78), food as reward (3, α = 0.66-0.69), child control (5, α = 0.49-0.70), modeling (4, α = 0.77-0.84), restriction for weight control (8, α = 0.70-0.82), restriction for health (4, α = 0.69-0.81), teaching about nutrition (3, α = 0.60-0.68), encourage balance and variety (4, α = 0.58-0.73), pressure to eat (4, α = 0.79), healthy environment (4, α = 0.75), involvement (3, α = 0.77)	Computer- assisted, self-administered	Parents of 1.5-8 year old children, primarily white and high income, also translated for use with a Norwegian population [88]
**Reinaerts (2007)** Social Influence on F&V Consumption [89]	63	mother/father modeling of F&V (4 individual items)	Self-administered paper survey	Parents of 4-12 year old children, Dutch population
**Stanton (2007)** Diet-Specific Social Support [90]	10	positive family support (5, α = 0.82)	Self-administered paper survey	Children aged 11-12 years old, rural population
**Vue (2007)** Individual and Environmental Influences on Calcium Intake [91]	36	independence (3, α = 0.67), parental expectations (2, α = 0.94), parental modeling (2, α = 0.81), family limitations (4, α = 0.61)	Self-administered paper survey	Children aged 10-13 years old, Hmong population
**Bryant (2008)** Healthy Home Survey [92]	113	food environment (8), eating practices (9), eating policies (11)	Phone interview	Parents of 3-8 year old children, primarily white and middle-upper income
**Burgess-Champoux (2008)** Determinants of Whole Grain Intake [93]	41	enabling behaviors (4, α = 0.82), role modeling (5, α = 0.63)	Not specified	Parents of 10-11 year old children, primarily white
**Byrd-Bredbenner (2008)** Food Decision Influencer [94]	67	food-related activities (10), food characteristics (10), family meals (12)	Self-administered paper survey	Parents of children 12 years or younger, primarily white
**Faith (2008)** Feeding Demands Questionnaire [95]	8	anger/frustration (4, α = 0.86), food amount demandingness (2, α = 0.86), and food type demandingness (2, α = 0.70)	Self-administered paper survey	Mothers of 3-7 year old children, twins, racially diverse
**Fulkerson (2008)** Family Meals [96]	24	family meal routines (7), family meal frequency (3), mealtime conflict (1), TV and eating (3), meal planning and preparation (3), frequency of making separate meals for children and adults (1)	Self-administered paper survey	Parents of 8-10 year old children, primarily white and college graduated
**Gatshall (2008)** Home Environment Survey [97]	126	parental role modeling of healthy eating (13, α = 0.83), parental policies to support healthy eating (11, α = 0.79)	Self-administered paper survey	Parents of 8-13 year old children, children were overweight or obese
**Haerens (2008)** Home Environment Related to Eating [98]	12	food rules (4), TV viewing (3)	Self-administered paper survey	Children aged 11-13 years old, Belgian population
**Haire-Joshu (2008)** High 5 for Kids [99]	15	coercive child feeding practices (4), modeling of F&V intake (1)	Phone interview	Parents of 1-6 year old children
**Kroller (2008)** Parental Feeding Strategies [100]	21	restriction (6, α = 0.75), monitoring (3, α = 0.93), pressure (3, α = 0.84), rewarding (4, α = 0.77), child control (3, α = 0.73), modeling (2, α = 0.77)	Self-administered paper survey	Mothers of 3-6 year old children, German population, lower income
**Spurrier (2008)** Physical and Nutritional Home Environment Inventory [101]	74	parental behaviors associated with food	In-person interview (with observation component)	Parents of 4-5 year old children, higher income
**Hendy (2009)** Parent Mealtime Action Scale [102]	31	snack limits (3, α = 0.81), positive persuasion (4, α = 0.75), daily F&V availability (3, α = 0.70), use of rewards (4, α = 0.65), insistence on eating (3, α = 0.68), snack modeling (3, α = 0.54), special meals (4, α = 0.45), fat reduction (3, α = 0.59), many foods choices (4, α = 0.42)	Self-administered paper survey	Parents of 3-10 year old children, most samples were primarily white
**Joyce (2009)** Parent Feeding Dimensions Questionnaire [103]	32	supportiveness (10, α = 0.81), structure (6, α = 0.72), coerciveness (10, α = 0.92), chaos (6, α = 0.80)	Self-administered paper survey	Parents of 4-8 year old children, primarily white
**Neumark-Sztainer (2009)** Ready Set ACTION [104]	62	parent weight talk (7, α = 0.82-0.85)	Child surveys administered by staff; self- administered paper survey for parents	Parents and children aged 9-12 years old, low income
**Pearson (2009)** Parental Modeling and Support [105]	7	parental modeling of eating behaviors (2), parental support for eating behaviors (2)	Self-administered surveys	Parents of 10-12 year old children, Australian population
**Corsini (2010)** Toddler Snack Food Feeding Questionnaire [106]	42	rules (10, α = 0.89, 0.85), flexibility (6, α = 0.87, 0.85), allow access (12, α = 0.88, 0.84)	Self-administered paper survey	Parents of 18 month-5 year old children, Australian population
**Dave (2010)** Home Nutrition Questionnaire [107]	25	parental practices that promote F&V intake 4, α = 0.77), parental role modeling (2, r = 0.75), amount of TV viewing (1)	Self-administered paper survey	Parents of 6-12 year old children, primarily Hispanic and low SES
**MacFarlane (2010)** Adolescent Perceptions of Parent Feeding Practices [108]	25	encouragement/modeling healthful eating (5, α = 0.74), negotiation (4, α = 0.67), pressure to eat disliked food (3, α = 0.66), pressure to eat when not hungry (3, α = 0.69), monitoring (2, α = 0.87)	Self-administered paper survey	Children aged 12-15 years old, Australian population
**McCurdy (2010)** Family Food Behavior Survey [109]	20	maternal control (5, α = 0.83), maternal presence (5, α = 0.76), child control (5, α = 0.80), organization (5, α = 0.73)	In-person interview	Parents of 2-11 year old children
**O’Connor (2010)** Food Parenting Practices [110]	33	teachable moments (5), practical methods (9), firm discipline (4), restriction of junk foods (5), enhanced availability/accessibility (10), across all scales α = 0.41-0.58	In-person interview	Parents of 3-5 year old children, racially and economically diverse
**Tremblay (2010)** Quebec Longitudinal Study of Development – Meal Interactions [111]	6	mealtime conflict (6, α = 0.55)	In-person interview	Parents of 4 year old children, population from Quebec
**Zeinstra (2010)** Parental Child-Feeding Strategies [112]	79	vegetable: positive information (4, α = 0.84), distraction (4, α = 0.67), choice (5, α = 0.70), negative atmosphere (4, α = 0.80), pressure (3, α = 0.76), taste masking (4, α = 0.62), habit (2, α = 0.42), extra veg (3, α = 0.59); fruit: negative atmosphere and pressure (8, α = 0.85), positive information (4, α = 0.82), distraction (3, α = 0.54), choice (5, α = 0.60); includes additional items not in scales	Self-administered paper survey	Parents of children 4-12 years old, Dutch population
**Berlin (2011)** Feeding Strategies [113]	32	mealtime structure (8, α = 0.75-0.82), consistent mealtime schedule (5, α = 0.84-0.87), child control of intake (8, α = 0.74-0.77), parent control of intake (6, α = 0.70-0.73), between meal grazing (3, α = 0.83-0.88), encourages clean plate (2, α = 0.83-0.89)	Self-administered computer survey	Parents of children 2-6 years old, primarily white
**Byrd-Bredbenner (2011)** Social Cognitive Theory Concepts [114]	39	self-regulation: sets goals - plans meals and shopping (3, α = 0.75), self-monitoring - uses food labels (3, α = 0.87), environmental structuring - TV during dinner (1)	Self-administered survey	Parents of children less than 12 years old, primarily white and moderate-high SES
**McIntosh (2011)** Family Meal Rituals [115]	12	dinner as a special family ritual (α = 0.77), a special family night (α = 0.87)	Telephone interview	Parents of children 9-15 years old, primarily white
**Moreno (2011)** Family Health Behavior [116]	27	parent behaviors (10, α = 0.85), mealtime routines (5, α = 0.77)	Self-administered paper survey	Parents of children 5-12 years old, racially and ethnically diverse
**Murashima (2011)** Parental Control Over Child Feeding [117,118]	24	high control (3, α = 0.70), high contingency (4, α = 0.79), child centered feeding (5, α = 0.66), nutrient dense food encouraging practice (2, α = 0.59), energy dense food discouraging practice (4, α = 0.74), mealtime behavior (3, α = 0.62), timing of meal (3, α = 0.64)	Self-administered paper survey	Parents of children 3-5 years old, low income
**Stifter (2011)** Baby’s Basic Needs [119]	13	food to soothe (13)	Self-administered paper survey	Parents of children 3-34 months old, higher income
**Anderson (2012)** Meals in Our Household [120]	60	structure of family meals (10, α = 0.66-0.73), use of food as a reward (6, α = 0.76-0.81), influence of child’s food preferences (3, α = 0.39-0.65)	Self-administered survey	Parents of children 3-11 years old
**Dave (2012)** Parental Social Support [121]	32	instrumental support (17, α = 0.87), positive encouragement (5, α = 0.76), negative role model (3, α = 0.83), discouragement to eat F&V (3, α = 0.78), and reinforcement (2, α = 0.50)	Self-administered paper survey	Parents of elementary-age children, primarily white
**Moore (2012)** West Virginia Healthy Lifestyle Act Evaluation [122]	82	parent actions regarding family diet (5)	Telephone interview	Parents of children 5-16 years old, primarily white
**Rigal (2012)** Feeding Style and Feeding Strategy [123]	38	authoritarian (7, α = 0.74), authoritative (7, α = 0.65), permissive (7, α = 0.70), coercion (6, α = 0.81), explanation (4, α = 0.72), contingency (4, α = 0.73), preference (3, α = 0.65)	Self-administered paper survey	Parents of children 20-36 months old, French population

1: Relevant scales must assess some aspect of food parenting practices. Only information on relevant scales was extracted. F&V = fruits and vegetables, FJV = fruits, juices, and vegetables, LFF = low-fat foods, veg = vegetables.

**Table 2:** Description of development and testing methods for parental feeding practice instruments

**Name of instrument**	**Score for concept.**	**Score/Methods for…**		**EFA factor loadings**	**Reliability evidence**	**Validity evidence**^**1**^
		**Item development**	**Item refinement**			
**Jensen (1983)** Family Routines Inventory [24,25]	4	3: interviews with families, literature review	mothers ranking of most important routines		Test-retest: r = 0.79	Construct Validity: total score on new survey was significantly correlated with the Family Environment Scale’s cohesion (rho = 0.35), organization (rho = 0.36), control (rho = 0.20, and conflict (rho = -0.18) scales.
**Stanek (1990)** Eating Environment [26]	2	2: expert opinion	expert review, pilot of survey			Construct Validity: child helps prepare food, child allowed to decide type of food eaten, use of small portions when introducing new foods, use discussion to persuade child to eat, leave child alone if refusing to eat, praise child for eating healthy were all associated with intake of foods from basic food groups (r = 0.18, p < .01).
**Seagren (1991)** Parents’ Behavior and Attitudes Toward their Children’s Food Intake [27]	4	3: expert opinion, observation and interviews in WIC clinics, literature review	expert review			Construct Validity: parents of overweight children were significantly less likely to report controlling the type of foods allowed for snacks, allowing sweets only after a healthy meal, encouraging child to eat all food on plate, and encouraging child to eat as much as they would like.
**Sherman (1992)** Maternal Feeding Practices Questionnaire [28]	3	2: pulled from existing surveys				Construct Validity: Pushier feeding practices was not significantly correlated with child weight.
**Davies (1993)** About Your Child’s Eating [29,30]	3	2: expert opinion	factor analysis	0.38-0.73		Construct Validity: AYCE factors correlated significantly and in expected directions with the Family Environment Scale factors (r = -0.9-0.39, p < .05 for all). Structural Validity: Final CFA model good fit, NFI = 0.87, NNFI = 0.91, R-CFI = 0.93, RMSR = 0.04, and SB-χ^2^/df = 1.75
**Crist (1994)** Behavioral Pediatrics Feeding Assessment Scale [31]	3	2: pulled from existing surveys	None		Test-retest: r = 0.83	
**Sallis (1995)** Study of Children’s Activity and Nutrition [32]	3	1				
**Koivisto (1996)** Mealtime Practices [33]	3	2: pulled from existing surveys	factor analysis	>0.30		
**Humphry (1997)** Feeding Stories [34]	4	3: interviews with parents, literature review	factor analysis	not reported	Test-retest: r = 0.68-0.90	
**De Bourdeaudhuij (1998)** Interactions Around Food [35,36]	4	2: pulled from existing surveys, open ended questions			Inter-rater: Pearson r = 0.02-0.49	Construct Validity: regression models showed that negative strategies was a significant predictor of child’s healthy food score (β = -0.17) and veg intake (β = -0.19); and obligation rules was a significant predictor of soda intake (β = -0.35).
**Golan (1998)** Family Eating and Activity Habits Questionnaire [37,38]	3	2: expert opinion, literature review	expert review, pilot of survey, factor analysis	not reported	Test-retest: r = 0.78 -0.90 Inter-rater: r = 0.81-0.94	Construct Validity: T-tests comparing scores from obese and normal-weight children showed that obese children have significantly higher scores on all scales and for total score (F(1,37) = 11.5).
**Hupkens (1998)** Food Rules [39]	3	2: qualitative study	pilot of survey			
**Fisher (1999)** Parental Restriction [40]	4	1				Construct Validity: Maternal use of restriction was significantly correlated with child selection of the restricted food (r = 0.41) and child weight for height (r = 0.42).
**Carper (2000)** Kids' Version of the Child Feeding Questionnaire [41]	4	2: pulled from existing survey			Inter-rater: pressure to eat was the only parent- reported variable that significantly predicted daughters’ perception (OR = 1.5)	Construct Validity: girls' perceived pressure to eat was significantly associated with dietary restraint (OR = 3.0), emotional disinhibition (OR = 3.2), and external disinhibition (OR = 3.0), and perception of restriction was significantly associated with external disinhibition (OR = 0.4).
**Cullen (2000)** Parent Food-Socialization Practices [43]	4	2: pulled from existing surveys, focus groups with parents	factor analysis	0.41-0.89	Test-retest: r = 0.61-0.89	Construct Validity: Dinner FJV preparation was significantly correlated with child juice intake (r = -0.35).
**Neumark-Sztainer (2000)** Project EAT [46-48]	4	3: pulled from existing surveys, expert opinion, focus groups with youth	poor test-retest or internal consistency		Test-retest: r = 0.54-0.70	Construct Validity: regression model showed that social support for healthy eating and family meal patterns were significant predictors of child F&V intake, but only had indirect effect through home F&V availability. Structural Validity: 2003: Final CFA model had good fit, factor loadings were 0.37-0.82, χ^2^ (347) = 3099, NFI = 0.99, RMSEA CI 0.043, 0.046.
**Ross (2000)** Family Unpredictability Scale [49]	4	3: expert opinion	expert review, factor analysis, pilot with parents	0.47-0.85		Construct Validity: meals was significantly correlated with other measures of family functioning (r = 0.18-0.31). CFA: Final higher order model. Structural Validity: final CFA higher order model had good fit, (df) χ^2^ = 87 (102.9), GFI = 0.90, CFI = 0.96, AGFI = 0.86, RMSEA = 0.04, AIC = 168.9, AIC-S = 240, AIC-I = 630.6.
**Baughcum (2001)** Preschooler Feeding Questionnaire [50]	4	2: pulled from existing surveys, expert opinion, focus groups with dieticians and mothers, literature review	factor analysis	0.49-0.82		Construct Validity: Scores on relevant factors were not significantly different between parents of normal vs. overweight children.
**Birch (2001)** Child Feeding Questionnaire [52-54]	4	2: pulled from existing survey, findings from previous research	factor analysis	0.37-0.95		Construct Validity: In sample 1, pressure to eat (r = -0.26) and restriction (r = 0.13) were significantly correlated with child weight. In sample 2, only responsibility (r = 0.20) was significantly correlated. In earlier study, controlling practices were significantly correlated with child’s ability to compensate for caloric density (r = 0.65). Structural Validity: Final CFA model in sample 1 had good fit, χ^2^ (229) = 419, CFI = 0.95, NNFI = 0.94, RMSEA = 0.04; final model in sample 2 confirmed with minor modifications, χ^2^ (227) = 309, CFI = 0.92, NNFI = 0.91, RMSEA = 0.05; final model in sample 3 confirmed after 3 items removed, χ^2^ (166) = 232, CFI = 0.91, NNFI = 0.89, RMSEA = 0.05. Criterion Validity: Mothers’ reported practices were not correlated with observed mealtime behaviors. Fathers’ reported pressure to eat was significantly correlated with observed use of pressure (0.36), prompting (0.65), and use of incentives (0.44); and reported restriction was significantly correlated with observed use of pressure (0.37) and use of incentives (0.47).
**Cullen (2001)** Family and Peer Influences on FJV Intake [61]	4	2: pulled from existing surveys, focus groups	factor analysis	0.43-0.85	Test-retest: r = 0.19-0.59	Construct Validity: Parent FJV modeling was significantly correlated with child intake of fruit (r = 0.18), juice (r = 0.14), total FJV (r = 0.20); and parent control was significantly correlated with child juice intake (r = 0.17).
**Tibbs (2001)** Parental Dietary Modeling Scale [62,63]	4	2: focus groups with parents, literature review				Construct Validity: Tibbs found that modeling was significantly associated with eating patterns (r = 0.48), low fat eating (r = -0.30), and F&V intake (r = 0.18). Moens found that parental modeling did not differ significantly between normal and overweight children,and parental modeling did not contribute to the prediction model snack intake.
**Tiggemann (2002)** Control Over Child Feeding [64]	4	1	factor analysis	0.53-0.83		Construct Validity: monitoring was significantly correlated with child BMI (r = 0.30) and BMI % (r = 0.33).
**Wardle (2002)** Parental Feeding Style Questionnaire [65]	4	3: pulled from existing surveys, interviews with mothers, literature review	cognitive interviews, pilot		Test-retest: r = 0.76-0.83	Construct Validity: Prompting/encouragement to eat was the only scale significantly correlated with child BMI (r = 0.19), and only significant for first-born twins.
**Bourcier (2003)** Eating for a Healthy Life – Strategies to Influence Eating Behavior [68]	3	2: pulled from existing surveys	factor analysis	not provided		Construct Validity: Reliance on self was a significant factor in model predicting child fat intake (B = -1.35, SE = 0.07); and pressure was a significant factor in the model predicting F&V intake (B = 1.44, SE = 0.04).
**Cullen (2004)** GEMS - Diet-Related Psychosocial Questionnaire [69]	3	2: pulled from existing surveys	factor analysis	0.37-0.73	Test-retest: ICC = 0.66-0.69	Construct Validity: low-fat food preparation practices was significantly associated with lower percent energy from fat (r = 0.23); high-fat food preparation practices was significantly associated with higher percent energy from fat (r = 0.24).
**Melgar-Quinonez (2004)** Child Feeding Strategies [70,71]	2	2: focus groups with parents	factor analysis	0.67-0.76		Construct Validity: Multivariate analysis did not find any of the scales to be associated with child overweight; however, child takes food from refrigerator or panty between meals was significantly associated with obesity (OR = 0.32)
**Vereecken (2004)** Food Parenting Practices [72,73]	4	2: discussions with parents, literature review				Construct Validity: permissiveness was significantly correlated with child intake of veg (r = -0.16), soda (r = 0.59), and sweets (r = 0.23). Pressure was significantly correlated with intake of veg (r = 0.15). Material reward was significantly correlated with intake of sweets (r = 0.19). Verbal praise was significantly correlated with intake of fruit (r = 0.16), veg (r = 0.20), and soda (r = -0.14). Negotiation was significantly correlated with intake of veg (r = 0.19). Encouragement was significantly correlated with intake of fruit (r = 0.22). Catering on demand was significantly correlated with intake of veg (r = -0.14), soda (r = 0.15), and sweets (r = 0.16). Full regression model found that permissiveness was a significant predictor of soda intake (OR = -8.81), material reward was a significant predictor of sweets intake (OR = 1.54), and praise was a significant predictor of veg intake (OR = 1.38).
**De Bourdeaudhuij (2005)** Pro Children Project [74]	4	3: expert opinion, focus groups with children, interviews with parents and staff, literature review	cognitive interviews, poor test-retest		Test-retest: r = 0.50-0.73	Construct Validity: active parent encouragement was significantly correlated with child intake of fruit (r = 0.17) and veg (r = 0.24); demand family rule was significantly correlated with child intake of fruit (r = 0.22) and veg (0.15); and allow family rule was significantly correlated with child intake of veg (r = 0.17).
**Horodynski (2005)** Child-Parent Mealtime Behavior Questionnaire [75]	3	2: pulled from existing survey	factor analysis	not reported		
**Hughes (2005)** Caregiver’s Feeding Style Questionnaire [6,76]	4	2: pulled from existing surveys, cognitive interviews, videotaped observations of mealtimes, literature review	factor analysis, low variability	0.30-0.65	Test-retest: ICC = 0.82-0.85	Construct Validity: parents with indulgent feeding style were more likely to have overweight children compared to authoritarian parents (F (3, 227) = 2.19, p < 0.04). Also noted significant main effects for feeding styles with the CFQ (F (9, 518) = 3.17) and the Parenting Dimensions Inventory (F (27, 602) = 2.26)
**Tripodi (2005)** Family Dietary Habits (part of the Italian National Institute of Nutrition) [78]	1	2: pulled from existing surveys				Construct Validity: parent behavior when child refuses to eat was significantly associated with child BMI (β = 0.86).
**Vereecken (2005)** Social and Environmental Influences on FJV Consumption [79]	4	2: pulled from existing surveys, open ended survey questions, literature review	team reviewed items from existing tools and reduced		Test-retest: ICC = 0.44-.071	Construct Validity: perceived parent behavior was significantly correlated with child intake of fruit (r = 0.30) and veg (r = 0.45); and permissiveness was significantly correlated with veg intake (0.15).
**Arredondo (2006)** Parenting Strategies for Eating and Activity Scale [80,81]	3	2: Pulled from existing surveys, focus groups with mothers	factor analysis	not provided		Construct Validity: Arredondo found that monitoring (β = 0.45), reinforcement (β = 0.32) and discipline (β = 0.20) were significantly associated with healthy eating; and monitoring (β = -0.17), reinforcement (β = -0.08) and control (β = 0.10) were significantly related to unhealthy eating. Noted some interactions with child gender. Larios found that control was significantly associated with child BMI (r = -0.21, p < 0.01). Also noted significant associations between PEAS scales and Birch’s CFQ scales. Structural Validity: In Arredondo, CFA model fit was good, χ^2^ (279) = 2.79, RMSEA = 0.06. In Larios, CFA final model fit was good, χ^2^ (282) = 1030.81, CFI = 0.89, IFI = 0.90, RMSEA = 0.06
**Ogden (2006)** Overt and Covert Control [82-84]	4	2: discussions with mothers, literature review	factor analysis	0.54-0.81		Construct Validity. overt control was significantly correlated with CFQ’s restriction (r = 0.27), pressure to eat (r = 0.46), and monitoring (r = 0.39); and covert control was also significantly associated with the 3 CFQ subscales (r = 0.42, 0.26, and 0.42). Regression models also showed that covert control predicted of unhealthy snack food and F&V intake, and overt control predicted F&V intake. Neither covert or overt control helped predict child BMI.
**de Moor (2007)** Management Techniques of Feeding Problems [85]	3	2: pulled from existing surveys	factor analysis	0.33-0.84		Construct Validity: positive behavioral support was significantly correlated with child pickiness (r = 0.47) and disturbing mealtime behavior (r = 0.46); as was negative behavioral support (r = 0.38 and 0.47) and general management technique (r = 0.17 and 0.28).
**Gray (2007)** Parental Attitudes around Feeding [86]	4	2: pulled from existing surveys, expert opinion, and literature review	expert review		Not reported	Construct Validity: parents of overweight or at risk for overweight children were significantly more likely to disagree with statement about encouraging the child to eat more.
**Musher-Eisenman (2007)** Comprehensive Feeding Practices Questionnaire [87]	4	3: pulled from existing surveys, open ended survey items, systematic review	factor analysis, eliminated items that were confusing or had no variability	0.31-0.95		Structural Validity: final CFA model fit was good, χ^2^ (1061) = 1580, CFI = 0.98, RMSEA = 0.057
**Reinaerts (2007)** Social Influence on F&V Consumption [89]	3	2: interviews with children and parents, literature review	factor analysis	0.66-0.91		Construct Validity: regression models showed that parent modeling of F&V intake was significant predictor child F&V intake (β = 0.0-0.34).
**Stanton (2007)** Diet-Specific Social Support [90]	3	2: pulled from existing surveys	factor analysis	0.52-0.81		Construct Validity: family support was a significant predictor of fiber intake (β = 0.23)
**Vue (2007)** Individual and Environmental Influences on Calcium Intake [91]	3	3: pulled from existing surveys, focus groups with children	factor analysis	0.41-0.71		Construct Validity: independence was significantly correlated with child intake of cheese (r = 0.24), parental expectations was significantly correlated with intake of soy milk (r = 0.21), parental modeling was significantly correlated with intake of soda (r = -0.24), OJ (r = -0.23), and dark green veg (r = -0.29), family limitations was significantly correlated with intake of soda (r = -0.19) and cheese (r = 0.31).
**Bryant (2008)** Healthy Home Survey [92]	3	2: pulled from existing surveys, expert opinion, literature review	expert review		Test-retest: percent agreement = 42.2-97.8, Kappa = 0.36-0.88, and ICC = 0.32-0.93	Criterion Validity: (only available for food environment items): percent agreement = 57.7-92.3, Kappa = 0.07-0.57.
**Burgess-Champoux (2008)** Determinants of Whole Grain Intake [93]	4	3: pulled from existing surveys, focus groups with parents, literature review	pilot of survey, factor analysis	0.46-0.89	Test-retest: Not reported for relevant scales	
Byrd-Bredbenner (2008) Food Decision Influencer [94]	4	2				Construct Validity: cluster analysis identified 4 clusters: (1) happy, healthy food involved mothers, (2) working, convenience driven mothers, (3) healthy, free of food price, taste, convenience, and advertising effects mothers, and (4) stressed, emotional eating, time-conscious mothers. Cluster 1 had significantly lower mother and child BMIs compared to other clusters.
**Faith (2008)** Feeding Demands Questionnaire [95]	3	2: expert opinion	factor analysis	0.78-0.89		Construct Validity: across 2 samples, total FEEDS score was significantly associated with CFQ’s monitoring (r = 0.30-0.36) and pressure to eat (r = 0.41-0.53); anger/frustration subscale was significantly associated w CFQ’s pressure to eat (r = 0.32-0.47); food amount demandingness was significantly associated with CFQ’s monitoring (r = 0.29-0.45), restriction (r = 0.24-0.26), and pressure to eat (r = 0.38-0.46); and food type demandingness was significantly associated with CFQ’s monitoring (r = 0.36-0.43). None of the scales were consistently associated with child BMI z score.
**Fulkerson (2008)** Family Meals [96]	4	2: pulled from existing surveys				
**Gattshall (2008)** Home Environment Survey [97]	3	2: pulled from existing surveys	factor analysis, item performance (low variability, extreme means or low correlation with scale)	not provided	Test-retest: ICC = 0.80-0.82 Inter-rater: ICC = 0.24-0.54;	Construct Validity: parental role modeling was significantly correlated with child’s intake of fruit (r = 0.21) and veg (r = 0.14); and parental policies was significantly correlated with child intake of fruit (0.28) and veg (0.36).
**Haerens (2008)** Home Environment Related to Eating [98]	2	2: pulled from existing surveys			Test-retest: ICC = 0.88-0.89 Inter-rater: r = 0.54-0.66	Construct Validity: food rules significantly contributed to prediction model for boys’ fat intake (β = 0.14) and girls’ fruit intake (β = -0.16); and TV viewing contributed to boys’ intake of soft drinks (β = 0.14) and fruit (β = -0.22) and girls’ intake of fat (β = 0.15) and fruit (β = -0.10).
**Haire-Joshu (2008)** High 5 for Kids [99]	2	2: pulled from existing surveys, focus groups			Test-retest: ICC = 0.50-0.66	Construct Validity: changes in parent modeling and use of non-coercive feeding did not predict changes in child F&V intake.
**Kroller (2008)** Parental Feeding Strategies [100]	3	2: pulled from existing surveys, expert opinion, interviews with mothers			Test-retest: r = 0.41-0.78	Construct Validity: regression model showed that pressure (β = 0.12) was a significant predictor of child intake of problematic foods; and child control (β = 0.24) and rewarding (β = -0.26) were significant predictors of child F&V intake.
**Spurrier (2008)** Physical and Nutritional Home Environment Inventory [101]	3	2: pulled from existing surveys				Construct Validity: portion size served, foods eaten in front of TV, acceptance of wasted food, reminding of child to ’eat up’, offering food as reward, and restriction of juice/high-fat and high-sugar foods/second helpings were significantly associated with child F&V intake; frequency of family meals, meals in front of TV, use of food to reward good behavior, and restriction of juice/carbonated beverage were significantly associated with child’s sweetened beverage intake; and use of food ’treats’ as reward for eating main meal, restriction of juice/high-fat and high-sugar foods, carbonated beverages, and snack/meals in front of TV were associated with child’s intake of non-core foods.
**Hendy (2009)** Parent Mealtime Action Scale [102]	4	2: pulled from existing surveys, literature review	factor analysis	0.42-0.87	Test-retest: r = 0.51-0.75 Inter-rater: r = 0.59-0.78	Construct Validity: positive persuasion (β = 0.07), daily F&V availability (β = 0.32), and special meals (β = -0.07) were significant predictors of child F&V intake; positive persuasion (β = 0.08), snack modeling (β = 0.17), fact reduction (β = -0.08), and many food choices (β = 0.08) were significant predictors of child snack intake; and positive persuasion (β = -0.08), insistence on eating (β = -0.12), snack modeling (β = 0.09), and fat reduction (β = 0.12) were significant predictors of child BMI%. Structural Validity: factor loading from CFAs in 3 samples ranged from 0.21-0.85, but no model fit indices were reported.
**Joyce (2009)** Parent Feeding Dimensions Questionnaire [103]	3	1	factor analysis referenced, but never published	not reported		Construct Validity: none of the feeding dimension were significantly correlated with child BMI; however, coerciveness was significantly correlated with child disinhibited eating (r = 0.16).
**Neumark-Sztainer (2009)** Ready Set ACTION [104]	2	2: pulled from existing surveys				
**Pearson (2009)** Parental Modeling and Support [105]	3	1				Construct Validity: parent modeling of breakfast was positively associated with F&V consumption in boys and girls (boys OR = 1.53, girls OR = 1.66).
**Corsini (2010)** Toddler Snack Food Feeding Questionnaire [106]	4	2: interviews with mothers	pilot with parents	0.21-0.82	Test-retest: ICC = 0.79-0.90	Construct Validity: rules and CFQ monitoring (0.40, 0.45), flexibility (ns, -0.32), and allow access (-0.21, -0.39) were significantly associated with CFQ monitoring; allow access was also significantly associated with CFQ restriction (0.28, ns). Also, rules was significantly associated with chip intake (-0.25); flexibility was significantly associated with intake of savory biscuits (0.20), sweet biscuits (0.18), chips (0.19), and high fat/sugar dairy (0.17); and allow access was significantly associated with intake of savory biscuits (0.38), sweet biscuits (0.42), cakes and pastries (0.28), chips (0.52), and high fat/sugar dairy (0.38). No significant associations with child BMI.
**Dave (2010)** Home Nutrition Questionnaire [107]	4	3: pulled from existing surveys, focus groups and interviews with mothers	cognitive interviews, factor analysis	0.58-0.87		Construct Validity: regression model showed that parental practices to promote F&V intake (β = 0.61) and role modeling (β = 0.34) were significant predictors of home F&V availability and accessibility.
**MacFarlane (2010)** Adolescent Perceptions of Parent Feeding Practices [108]	2	2: pulled from existing surveys	factor analysis	0.51-0.89		Construct Validity: significant score differences between parents who were concerned vs. not concerned with child weight were observed for negotiation (-0.17 vs. 0.06, p < 0.001) and pressure to eat disliked food items (-0.08 vs. 0.03, p = 0.05).
**McCurdy (2010)** Family Food Behavior Survey [109]	4	2: pulled from existing surveys	cognitive interviews, factor analysis	0.43-0.90	Test-retest: ICC > 0.65	Construct Validity: all scales were significantly correlated with at least one other scale. Child choice was significantly correlated with maternal control (-0.47) and organization (0.34); and maternal control was significantly associated with maternal presence (0.34). Mothers with overweight children also had higher scores on maternal control (t(23) = 2.06, p = 0.052), but only at time 1. Mothers of normal weight children had higher scores on maternal presence (t(19) = -2.85, p = 0.01), but only at time 2.
**O’Connor (2010)** Food Parenting Practices [110]	4	3: expert opinion, focus groups with parents				Construct Validity: practical methods was significantly correlated with child F&V intake (r = 0.08), and firm discipline was significantly associated with child BMI z-score (r = -0.14). Neither parent practice categories or clusters contributed significantly to the model of child F&V.
**Tremblay (2010)** Quebec Longitudinal Study of Development – Meal Interactions [111]	3	1				Construct Validity: in boys, meal conflict had a direct effect on child body weight (more conflicts, higher body weight) and healthy eating (more conflicts, healthier eating). In girls, meal conflict had a direct effect on healthy eating (more conflicts, healthier eating). Structural validity: For boys, Nχ^2^ = 2.48, CFI = 0.94, SRMR = 0.04, RMSEA = 0.05; and for girls, Nχ^2^ = 1.68, CFI = 0.97, SRMR = 0.03, RMSEA = 0.03.
**Zeinstra (2010)** Parental Child-Feeding Strategies [112]	3	2: pulled from existing surveys	factor analysis, pilot with parents	0.49-0.82		Construct Validity: regression models showed that choice (β = 0.28), distraction (β = -0.13), negative atmosphere (β = -0.19), pressure (β = 0.21), and positive info (β = -0.13) were significant predictors of child vegetable intake; and choice (β = 0.17) and negative atmosphere and pressure (β = -0.12) were significant predictors of child fruit intake. Correlations were also observed between CFQ scales on this new survey.
**Berlin (2011)** Feeding Strategies [113]	3	2: expert opinion	factor analysis, agreement between project team members ratings of items’ potential fit with constructs	0.33-0.89		Construct Validity: r = -0.43-0.46, significant correlations were observed between: across 2 samples, mealtime structure was significantly correlated with meal schedule (0.38, 0.45), child control of intake (-0.16, 0.12), parent control of intake (ns, 0.11), and between meal grazing (-0.27, -0.37); meal schedule was significantly correlated with parent control (ns, 0.14), between meal grazing (-0.28, -0.30), and clean plate (ns, 0.12); child control of intake was significantly correlated with parent control (ns, -0.34), between meal grazing (0.18, 0.22), and clean plate (ns, -0.27); parent control of intake was significantly correlated with between meal grazing (ns, -0.19), and clean plate (0.46, 0.38); between meal grazing was significantly correlated with clean plate (ns, -0.15). All but encourages clean plate were significantly correlated with one or more scales from the About Your Child’s Eating Scale. Structural Validity: For the community sample, SB χ = 980.43, df = 448, CFI = 0.91, RMSEA = 0.064 (90% confidence interval: 0.058-0.069).
**Byrd-Bredbenner (2011)** Social Cognitive Theory Concepts [114]	4	2: pulled from existing surveys, expert opinion, literature review				Construct Validity: mothers scoring in the lowest tertile for plans meals had significantly higher BMIs compared to those in the highest tertile (p = 0.0031, F = 3.531).
**McIntosh (2011)** Family Meal Rituals [115]	3	2: pulled from existing surveys	factor analysis	not reported		Construct Validity: logistic regression showed that father’s perception of the family dinner as an important family ritual was a significant predictor of use of fast-food restaurants (OR = 0.39).
**Moreno (2011)** Family Health Behavior [116]	2	2: pulled from existing surveys, expert opinion, responses to caregiver survey, literature review	expert review, factor analysis, item performance	0.43-0.75	Test-retest: ICC = 0.75-0.77	Construct Validity: binary logistic regression showed that for every point increase in the total score, there was a 3.9% decrease in likelihood of child being overweight or obese (OR = 0.92, p < 0.01). However, bivariate correlations did not show significant associations between relevant scales (parent behavior, mealtime routines) and child zBMI.
**Murashima (2011)** Parental Control Over Child Feeding [117,118]	4	3: pulled from existing surveys, expert opinion	expert review, cognitive interviews, factor analysis	0.45-0.83	Test-retest: r = 0.45-0.85	Construct Validity: high control (-0.14) and high contingency (-0.13) were significantly associated with child BMI; child centered strategies (0.20), encouraging nutrient dense foods (0.26), and timing of meals (-0.12) were significantly associated with intake of nutrient dense foods; and encouraging nutrient-dense foods (-0.12) and discouraging energy dense foods (0.26) were significantly associated with intake of energy dense foods. Structural Validity: χ^2^ = 292, df = 179, p < 0.05, CFI = 0.927, RMSEA = 0.044 (after timing of meals was removed).
**Stifter (2011)** Baby’s Basic Needs [119]	3	1				Construct Validity: feeding to soothe was significantly correlated with pressuring (0.23) and indulgent (0.23) styles; and interaction of using food to soothe child and child negativity was a significant predictor of child BMI z-score (p = 0.012).
**Anderson (2012)** Meals in Our Household [120]	4	2: pulled from existing surveys, expert opinion, literature review	low item correlations were used to remove items from scales		Test-retest: r = 0.80-0.95	Construct Validity: across 2 samples, family meals was significantly correlated with problem behaviors (-0.51, -0.38), parental concern (-0.29, -0.52), food as a reward (-0.21, ns), and spousal stress (-0.35, -0.23); food as a reward was significantly correlated with problem behaviors (0.33, 0.52), parental concern (0.33, 0.46), spousal stress (0.31, 0.46), and child influence (0.24, 0.35); and child influence was significantly correlated with problem behaviors (0.31, 0.48), parental concern (0.36, 0.49), and spousal stress (0.47, 0.38).
**Dave (2012)** Parental Social Support [121]	4	2: pulled from existing surveys, expert opinion, literature review	factor analysis	0.42-0.99	Test-retest: r = 0.56-0.94	Construct Validity: instrumental support (0.25) and positive encouragement (0.15) were significantly associated with F&V availability; and instrumental support (0.45), positive encouragement (0.29), and reinforcement (0.19) were significantly associated with F&V accessibility.
**Moore (2012)** West Virginia Healthy Lifestyle Act Evaluation [122]	1	2: pulled from existing surveys				Construct Validity: logistic regression showed that parents who were concerned with their child’s weight were significantly more likely to report trying to change their family’s diet to make it healthier, put their child on a diet, and have their child skip meals or snacks.
**Rigal (2012)** Feeding Style and Feeding Strategy [123]	4	2: interviews with mothers	factor analysis	0.57-0.85		Construct Validity: partial least squares regression model identified several factors with regression coefficients >0.1 including: permissive style, contingency strategies, preference strategies, and coercion strategies. Structural Validity: χ^2^(173) = 442.39, CFI = 0.88, NNFI = 0.86, RMSEA = 0.055.

1: Significance of all findings is based on p ≤ 0.05. CFQ = Child Feeding Questionnaire.

Among the food parenting practice questionnaires included in this review, final surveys had between 6 and 221 items (44 items on average). While all instruments had at least one relevant scale or categorical grouping of items to assess parent food practices, items within these scales or categories represented less than half of the items in the instrument. These instruments had between 2 and 76 relevant items (19 relevant items on average) and anywhere between 1 and 12 relevant scales or categories (3 to 4 on average). As described in Table 1, the constructs measured varied widely from one instrument to another. Often instruments focused on measuring either controlling feeding practices or supportive and encouraging feeding practices.

### Conceptualization of instrument’s purpose

The terms used to describe what the instruments were intended to measure varied, in part, on the background from which the instrument arose. In addition to parent food practices, common terms included: parent-child feeding practices, feeding strategies, feeding style, feeding dimensions, feeding relationship, mealtime environment, mealtime actions, mealtime interactions, parent-child mealtime behaviors, food socialization practices, home food environment, amongst others. Each of these terms has a slightly different definition; however, all of the instruments included items that measured parent food practices. Despite differences in terms, 87% did conceptualize and define what they intended to measure, with 35 instruments receiving the maximum score of 4 and 27 instruments receiving a 3 for conceptualization. Just under half (33 of 71) noted a theoretical basis for the development of their instrument. More commonly referenced theories included: Social Cognitive Theory (n = 12) [43,46,61,62,91,93,98,102],[104,107,114,121], Social Ecologic Framework (n = 3) [97,98,101], Theory of Planned Behavior (n = 3) [35,74,98], Social Learning Theory (n = 2) [37,82], Costanzo and Woody’s Domain Specific Parenting or Baumrind’s Parenting Styles (n = 4) [6,52,64,123], and Satter’s model of the feeding relationship (n = 3) [27,29,113].

### Development of item pool

The processes used to develop a pool of items varied widely. Only 14 (20%) received the maximum score of 3, indicating a fully systematic process was employed. Common methods employed for item development included: pulled or modified items from existing instruments (n = 44), extrapolation from qualitative formative data such as focus groups or interviews (n = 36), created items based on a review of the literature (n = 22), expert guidance (n = 19), or some combination of methods (n = 33). Seven instruments had no description regarding how items were created.

### Refinement of item pool

About one third (n = 24) of the instruments identified in this review did not report any attempts to refine the pool of items once created. Among those who did attempt to refine their pool of items, factor analysis was the most commonly used method (n = 36). Those who employed factor analyses generally used widely accepted criteria for cut-offs for factor loadings and cross loadings. Only 16 instruments had items reviewed by experts to assess content validity, and only 30 piloted the instrument or conducted cognitive interviews to assess clarity of items and face validity. Item performance was also noted as a means to reduce the item pool for 7 instruments.

### Reliability

Some form of reliability was reported for a majority of instruments (n = 57 or 80%). Internal consistency was the most common form of reliability reported (n = 56). Generally those that employed such methods retained only those scales that met generally established cut-off criteria with 38 reporting Cronbach’s alphas of at least 0.6 or higher. None of these 38 instruments had alphas greater than 0.9 for all scales, only 5 had alphas consistently above 0.8, and an additional 23 had alphas consistently above 0.7. Test-retest was reported for 27 instruments, typically using a 1-3 week interval. The two notable exceptions were the GEMS’ Diet-Related Psychosocial Questionnaire [69], which administered test-retest over a 12-week period (during which time there was also an intervention delivered); and the Behavioral Pediatrics Feeding Assessment Scale [31], which administered test-retest over a 2 year interval. Correlations reported for test-retest were generally acceptable (>0.6) for most scales within a given instrument. However, when looking at test-retest correlations of all scales on a given instrument only 5 had correlations for all scales above 0.8; 6 additional had correlations >0.7; and 5 more had correlations >0.6. Inter-rater reliability was reported for 6 instruments, but only 1 instrument reported that all correlations were >0.8, the remaining 5 included correlations less than 0.6 for at least one scale.

### Validity

The majority of instruments (n = 61 or 86%) reported some type of validity evidence. Construct validity was by far the most common type of validity evidence evaluated (n = 59), often testing for relationships between food parenting practices and child diet or child weight. Most instruments had one or more scales that were significantly associated with one of these outcomes; however, correlations were generally in the range of 0.15-0.45. While all papers including this type of evidence were given credit for evaluating construct validity, these tests were not always presented as construct validity within the articles. Confirmatory factor analysis was reported for only 10 instruments. Those that did attempt to explore structural validity were generally successful with only minor modifications to their original model. Only two studies attempted to establish criterion validity.

### Responsiveness

The Family Eating and Activity Habits Questionnaire [37] was the only paper that formally assessed the instruments’ responsiveness to treatment results. The questionnaire was administered to families taking part in a weight loss program both at baseline and follow-up. Changes in questionnaire scores as well as changes in weight were observed in the intervention group, and weight loss in the child was highly correlated with improvement in the questionnaire score.

### Completeness of development process

Ideally, instrument development would involve all 6 components described thus far: (1) clear conceptualization of what the instrument is intended to measure, (2) systematic process for developing item pool, (3) refinement of the item pool (through at least one method: factor analysis, expert review, cognitive interviews, and/or piloting), (4) some type of reliability testing (inter-rater, test-retest, and/or internal consistency), (5) at least one type of validity testing, and (6) responsiveness or stability testing. On average, instruments reported only 2 or 3 of these 6 steps (range: 0 to 4).

## Discussion

In the current review, 71 instruments were identified that included assessment of parent food practices. The quality of processes used and reported for instrument development varied widely, but there are instruments that demonstrate reasonably thorough development work. The quality assessment of the 71 instruments in this review highlights many key lessons that should inform future research in the areas of conceptualization of constructs, development and refinement of the item pool, collection of multiple types of reliability and validity evidence, and planning for responsiveness or stability testing.

### Conceptualization of constructs

Parent food practices is a rapidly growing area of research that would benefit greatly from a common conceptual model. The content map (Figure 2) represents an initial effort to capture relevant constructs that should be included in this conceptual model. It served as a useful guide for the current review and may help inform future work to develop a conceptual model. Consensus is required in order to develop a clear conceptual model including an indication of what constructs should be included and how those constructs should be defined. The current lack of consensus has resulted in scales from different instruments that may share similar names, but include items measuring very different behaviors. Further, other instruments may include similar items, but employ different names for their scales. For example, the Restriction subscale from the Child Feeding Questionnaire [52] includes items about ensuring the child does not eat too many sweets or high fat foods and items about guiding and regulating child’s intake of certain foods – both of which reflect how “restriction” is typically defined. However, this subscale also includes items regarding offering sweets as a reward for good behavior, which other measures call “instrumental feeding” [65]. Researchers working in this field need consensus and a clear conceptual model based on current knowledge. Future research can then expand upon or clarify components of the model.

The use of theory to guide instrument development helps ensure clear conceptualization of all relevant constructs. Unfortunately, only half of developers noted a theoretical basis for their instruments. Social Cognitive Theory (SCT) [124] and the Social Ecologic Framework [125,126] were two of the most commonly referenced theories, both of which recognize the influence that the environment, and the shared environment in particular, has on behavior. A number of instruments originated from family psychology, using theories about Social Learning Theory [127], Parenting Dimensions [128-130], and Domain Specific Parenting [131] to guide development of their instruments. These theories generally recognize that parents play a central role in the socialization of their children and hence the behaviors that a child adopts, including eating behaviors. All of the theories provided useful guidance to instrument development, and should be considered in efforts to develop a conceptual model for parent food practices.

### Development and refinement of the item pool

Ideally, development and refinement of the item pool uses a systematic approach that involves multiple methods and allows for multiple iterations. Consulting the current literature is a good starting point, but less than one third of instruments reported reviewing the literature as part of their process. Many instruments reported pulling items from existing instruments, but it is unknown how systematically existing instruments were reviewed before selecting which instruments and items to use for the new measure. Development of a new measure should also address gaps in measurement, creating items and scales for constructs not being measured by current instruments. Informed processes are needed to guide creation of new questions. Qualitative data (e.g., focus groups and interviews) can provide such a resource, but less than half of instruments reported drawing on such data sources. Once an initial item pool is created, it is also important to evaluate and refine that item pool; however, over a third of instruments reported no details on item refinement. Among those that did, factor analysis was the most common strategy employed. Expert review, cognitive interviews and piloting are important steps for ensuring complete content coverage and inclusion of items that are easily interpreted by the target audience. However, very few instruments reported assessment of content or face validity. The development article for the Comprehensive Feeding Practices Questionnaire [87] provides a useful example of a thorough and iterative process combining multiple strategies to generate and refine an item pool. To create an initial item pool, these researchers drew items from most widely used instruments, adapted items from adult measures where no existing items existed and reviewed the literature to gather information about additional constructs. The original item pool was piloted and factor analysis used to identify constructs needing additional items. Then, open-ended questions were given to another sample of parents to help generate these additional items. Future researchers interested in instrument development should aim to adopt similar methodologies and incorporate multiple strategies into their own plans for developing and refining the item pool for their new instruments.

### Reliability and validity evidence

While almost all instruments received credit for performing some evaluation of reliability (80%) or validity (86%), there was clear reliance on more statistical approaches using data collected from a single time point for supplying such evidence. For reliability, many instruments presented only a Cronbach’s alpha. While this provides information on how well items within a scale group together, it does not provide evidence of repeatability. Assessment of test-retest and inter-rater reliability provides this type of evidence, but requires more investment in data collection. Not surprisingly, few instruments included these latter types of evaluation. Similarly, most validity evidence came from assessment of construct validity. Structural validity and criterion validity require greater investment in data collection to administer the instrument in multiple samples or to collect a gold standard measure. Use of these latter types of validity was limited. A thorough assessment of reliability and validity should include multiple strategies for each, which will require researchers to devote more time to instrument development by collecting data across multiple time points or in multiple samples or incorporating use of a gold standard.

### Responsiveness testing

The area of instrument development that clearly needs the most attention is instrument responsiveness. Researchers seeking to evaluate interventions need evidence regarding the level of change that these instruments are able to detect. This type of information is essential when trying to calculate power and sample size needed for a study. While many of these instruments have indeed been used in studies to evaluate interventions, responsiveness testing is almost never reported as part of the development.

### Additional issues

Another important consideration when selecting an instrument is its relevance for the target population. The feeding relationship changes as children get older, and hence the feeding practices parents employ change as well. At younger ages, children are more dependent on their parents to provide food choices. As they get older, they become more independent and peers are thought to exert a greater influence on eating habits [132], which may in turn influence the feeding approaches parents employ. All of the instruments included in this review were developed for families with children between the ages of 2 and 12 years old. Similarly, parent food practices may vary across different cultural groups [44,51]. Some practices may appear to be detrimental to healthy eating habits in certain populations, but those same practices are found to be protective in others. For this reason, Table 1 describes the population in which each instrument was tested.

### Limitations

Authors of this review provided a comprehensive inventory and assessment of existing measures of parent food practices. However, the current review is limited to instruments developed for families with children 2-12 years old. Additional instruments that were developed for families with adolescent children are not included. We limited this review to younger children because the parents and the home environment are the predominant influence on child eating behaviors at this age. Similarly, this review is limited to articles written in English. While it includes instruments that were developed in other languages, there are additional non-English instruments that have undoubtedly been left out of the current inventory. The results focus on presenting the primary development articles for each of the identified instruments; however, many of these instruments have been used in later studies with different populations. During the review process, 244 articles were identified that described studies in which existing instruments were used. Some of these may provide additional information about construct validity (e.g., association with child diet or weight), but were not included or summarized here. However, articles in which there was clear development work to adapt and evaluate scales for new populations are captured in Table 1. Also, no attempt was made to provide an overall quality scores for each instrument. To be truly informative, a scoring rubric would need to take into account not just attempts to complete the various development steps, but also the appropriateness of tests used, and the significance of the outcomes across factors measured within an instrument. Such a scoring tool is expected to be complex and is not yet available at the time of writing. Therefore, the authors have summarized the development work that has been done, the reliability and validity evidence reported, and well-accepted criteria for assessing those results. Readers are thus able to judge for themselves the strength of the evidence in light of other factors.

## Conclusions

This review was able to identify 71 different measures of parent food practices. However, these existing instruments measure a variety of different constructs. Additionally, the rigor with which they were developed varied widely. Ideally, instrument development and evaluation are multi-staged processes that require time and patience. Researchers or practitioners who do not have the resources to dedicate to instrument development should be encouraged to look for existing instruments that measure the specific constructs needed for their study. Future work should focus on further evaluation of appropriate instruments where possible. Undoubtedly, new instruments will need to be developed; however, this future development work should consider the lessons learned from the current review and to consider all stages of development needed to create a valid and reliable measure.

## Competing interests

The authors declare that they have no competing interests.

## Authors’ contributions

All authors have made substantial contributions to the design, acquisition of data, and summarization of findings, and all have been involved in writing of the manuscript and given final approval of the version to be published. AV performed the original search and compiled the database of articles. AV, RT and MB reviewed titles and abstracts, and AV and RT applied inclusion and exclusion criteria to narrow the remaining pool. AV, RT, MB and DW all participated in the review of final articles, development of the quality assessment protocol, and use of this protocol to evaluate each instrument identified. AV summarized results and discussed findings with RT, MB and DW to develop structure for paper and tables and identify key messages for the discussion. All authors participated in the writing and editing of the final manuscript.

## Supplementary Material

Additional file 1 Search Terms for Diet and Feeding Environment. Click here for file
