# Supplementary material for: Measuring parent food practices: a systematic review of existing measures and examination of instruments
Source: Int J Behav Nutr Phys Act. 2013 May 20;10:61. doi: 10.1186/1479-5868-10-61 (PMC3681578; doi:10.1186/1479-5868-10-61)
Supplement: Additional file 1 — Search Terms for Diet and Feeding Environment. [file 1479-5868-10-61-S1.doc]

**Search Terms for Diet and Feeding Environment**

**Original search:**

(eating OR eating behavior OR eating behaviors OR diet OR diet behavior OR diet behaviors OR dietary behavior OR dietary behaviors OR meal OR meals OR breakfast OR dinner OR snack OR snacks OR snacking OR fruit OR fruits OR vegetable OR vegetables OR feeding OR feeding behavior OR feeding behaviors OR feeding practice OR feeding practices OR feeding style OR feeding styles OR feeding skills OR food environment OR eating environment OR cooking space OR cooking equipment OR cooking OR cooking practices OR food preparation OR cooking ability OR cooking skills OR dining area OR eating area OR eating location OR eating space OR kitchen table OR dining table OR meal structure OR mealtime OR mealtimes OR family meal OR family meals OR food availability OR food accessibility OR food storage OR food rules OR food policies OR food limits OR food monitoring OR food restriction OR pressure to eat OR food as reward OR non-nutritive feeding OR eating distractions OR role model OR role modeling OR parent modeling OR parent role model)

AND

(home environment OR family environment OR family OR family characteristics OR parent OR parents OR parent-child relations OR mother OR mothers OR father OR fathers OR parenting OR parenting style OR parenting styles OR parenting practices OR parenting skills OR parent behavior OR parent behaviors OR authoritarian OR authoritarian parenting OR authoritative OR authoritative parenting OR permissive parenting OR childrearing practices OR parental involvement)

NOT

(breastfeeding OR breast milk OR introduction of solids OR diarrhea OR rehydration OR dehydration OR eating disorder OR eating disorders OR cerebral palsy OR feeding tubes OR enteral nutrition OR cystic fibrosis OR dental carries OR oral hygiene OR allergy OR allergies OR virus OR viruses OR infection OR parasite OR mouse OR mice OR rat OR rats OR fish OR animal OR animals OR plant OR plants OR zoology) and English Language

LIMITS: English language

**Updated search:**

(“feeding behavior” OR “feeding practice” OR “feeding style” OR “feeding skill” OR “feeding dimension” OR “feeding relationship” OR “mealtime action” OR “mealtime interaction” OR “mealtime environment” OR “mealtime behavior” OR “food environment” OR “eating environment” OR cooking OR “food preparation” OR “meal structure” OR “family meal” OR “pressure to eat” OR “food as reward” OR “non-nutritive feeding” OR “eating distractions”)

OR

((feeding OR food OR eating OR diet OR meal) AND (socialization OR rule OR policy OR limit OR monitoring OR restriction OR reward OR model))

AND

(home OR family OR parent OR mother OR father OR child)

AND

(development OR psychometric OR reliability OR validity OR instrument OR scale OR “factor analysis”)

NOT

(breastfeeding OR “breast milk” OR “introduction of solids” OR diarrhea OR rehydration OR dehydration OR “eating disorder” OR “disordered eating” OR “cerebral palsy” OR “feeding tube” OR “enteral nutrition” OR “cystic fibrosis” OR “dental carries” OR “oral hygiene” OR allergy OR allergies OR virus OR infection OR parasite OR mouse OR mice OR rat OR fish OR animal OR plant OR zoology)

LIMITS: English language
